# Temporal trends in clinical and inflammatory features of Kawasaki disease across the COVID-19 pandemic: a single-center experience from Turkey

**DOI:** 10.3389/fped.2026.1871138

**Published:** 2026-07-15

**Authors:** Gamze Vuran, Murat Muhtar Yılmazer

**Affiliations:** Department of Paediatric Cardiology, University of Health Sciences, İzmir Dr. Behçet Uz Paediatric Diseases and Surgery Training and Research Hospital, İzmir, Türkiye

**Keywords:** clinical features, COVID-19, inflammation, Kawasaki disease, post-pandemic

## Abstract

**Background:**

The COVID-19 pandemic has been associated with changes in the epidemiology and clinical presentation of pediatric inflammatory diseases, including Kawasaki disease (KD). However, data comparing Kawasaki disease characteristics across pre-pandemic, pandemic, and post-pandemic periods remain limited.

**Methods:**

This retrospective single-center study included children diagnosed with KD between January 2015 and February 2024. Patients were classified as pre-pandemic (2015–2019), pandemic (2020–2021), or post-pandemic (2022–2024). Demographic characteristics, clinical features, laboratory findings, treatment response, and echocardiographic outcomes were compared among periods.

**Results:**

Eighty-seven patients were analyzed (pre-pandemic *n* = 46, pandemic *n* = 22, post-pandemic *n* = 19). During the pandemic period, C-reactive protein and erythrocyte sedimentation rate were significantly higher, thrombocytosis was more frequent, and cervical lymphadenopathy was less common. In the post-pandemic period, time to diagnosis was shorter, the number of clinical findings at presentation was higher, and elevations in liver transaminases were more frequent. Rates of intravenous immunoglobulin resistance, length of hospital stay, coronary artery involvement, and valvular regurgitation were similar across periods.

**Conclusion:**

The COVID-19 pandemic was associated with changes in the inflammatory and clinical expression of Kawasaki disease without clear differences in cardiac outcomes, suggesting a greater effect on disease phenotype than on overall disease course; however, these findings should be interpreted in the context of the retrospective design and limited sample size.

## Introduction

Kawasaki disease (KD) is an acute, self-limited febrile vasculitis of childhood. Cardiac involvement, particularly coronary artery aneurysm or dilatation, makes Kawasaki disease the leading cause of acquired heart disease in children in developed countries (1). Although its exact etiology remains unclear, epidemiologic patterns related to incidence, age distribution, and seasonality suggest an infectious trigger (2–4).

The coronavirus disease 2019 (COVID-19) pandemic, caused by severe acute respiratory syndrome coronavirus 2 (SARS-CoV-2), spread globally in 2020 and substantially altered the epidemiology of pediatric infectious and inflammatory diseases. Early reports from Italy and France described a 30-fold and fourfold increase, respectively, in children meeting KD diagnostic criteria, suggesting a potential link between SARS-CoV-2 infection and a KD-like inflammatory phenotype (5, 6). In May 2020, multisystem inflammatory syndrome in children (MIS-C) was identified, sharing overlapping features with KD but typically presenting with higher inflammatory burden and more severe clinical manifestations.

In contrast, several subsequent studies demonstrated a decline in KD-related hospitalizations during the early pandemic period (7), indicating heterogeneous regional trends. Despite multiple international reports (8–10), data regarding clinical and phenotypic changes in KD across the pandemic period remain limited in many regions.

Therefore, this study aimed to evaluate temporal changes in clinical phenotype and coronary involvement among children with Kawasaki disease across the COVID-19 pandemic, in a cohort specifically excluding cases of multisystem inflammatory syndrome in children (MIS-C).

## Methods

### Study design and population

This retrospective single-center study included consecutive children diagnosed with Kawasaki disease and followed in the Departments of Pediatrics and Pediatric Cardiology at our institution between January 2015 and February 2024. Patients were categorized according to date of diagnosis into three periods: pre-pandemic (2015–2019), pandemic (2020–2021), and post-pandemic (2022–2024).

### Diagnostic criteria and exclusion of MIS-C

Complete and incomplete Kawasaki disease were diagnosed according to the 2017 American Heart Association criteria. During the pandemic period, the medical records of all eligible patients were retrospectively reviewed to minimize potential diagnostic overlap with Multisystem Inflammatory Syndrome in Children. Patients with a final diagnosis of MIS-C, positive SARS-CoV-2 PCR testing at presentation, or clinical/laboratory findings considered more consistent with MIS-C were excluded.

SARS-CoV-2 PCR testing was performed in all 22 patients diagnosed during the pandemic period, and all results were negative. SARS-CoV-2 serology was additionally performed in six patients (three diagnosed during the final quarter of 2020 and all three patients diagnosed in 2021, corresponding to the period of highest MIS-C awareness in Turkey). Serologic testing was selectively obtained in patients with clinical features raising concern for MIS-C, including multisystem involvement (renal, gastrointestinal, respiratory, or neurological manifestations) together with markedly elevated inflammatory or coagulation markers (e.g., ferritin, D-dimer, fibrinogen), lymphopenia, or thrombocytopenia. Serologic testing became available at our institution in late 2020; prior to that time, diagnostic evaluation relied on PCR testing and clinical assessment. All serologic results were negative, and none of these patients fulfilled MIS-C diagnostic criteria.

### Ethics

The study was approved by the Clinical Research Ethics Committee of the University of Health Sciences, İzmir Dr. Behçet Uz Pediatric Diseases and Surgery Training and Research Hospital and conducted in accordance with the Declaration of Helsinki.

### Definitions and data collection

Fever duration before diagnosis was defined as the number of days from fever onset to establishment of Kawasaki disease diagnosis. Day to diagnosis was defined as the interval between hospital presentation and confirmation of diagnosis. Laboratory variables were recorded from the earliest available blood samples obtained during the initial diagnostic evaluation before intravenous immunoglobulin treatment. No laboratory values included in the analysis were obtained after IVIG administration. All variables included in the study were routinely collected as part of standard clinical care, and no missing data were present for any demographic, clinical, laboratory, echocardiographic, or treatment-response variable included in the analyses.

Laboratory definitions followed AHA recommendations and institutional reference standards. Thrombocytosis was defined as a platelet count ≥450,000/mm^3^ and hypoalbuminemia as a serum albumin level <3.0 g/dL at diagnosis. Anemia was defined according to age-adjusted reference values. Elevation of liver transaminases was defined as AST or ALT values >40 IU/L based on institutional laboratory reference limits.

### Echocardiography

Transthoracic echocardiography was performed at diagnosis, during the first week, at four weeks, and thereafter as clinically indicated. Coronary artery dimensions of the left main coronary artery, right coronary artery, and left anterior descending artery were measured, and Z-scores were calculated using the Boston nomogram with body surface area derived according to the Haycock formula. Coronary artery involvement was classified according to the 2017 AHA guidelines. All echocardiographic examinations were performed by the same group of experienced pediatric cardiologists using standardized echocardiographic acquisition techniques and coronary artery measurement methods consistent with American Heart Association recommendations throughout the study period.

### Treatment

All patients received intravenous immunoglobulin (IVIG) at a dose of 2 g/kg and high-dose acetylsalicylic acid during the acute phase, followed by low-dose therapy. IVIG resistance was defined as persistent or recurrent fever ≥36 h after completion of the initial infusion. Management of coronary aneurysms followed AHA recommendations.

### Statistical analysis

Statistical analyses were performed using IBM SPSS Statistics version 27. Continuous variables were analyzed using ANOVA or Kruskal–Wallis tests as appropriate. When overall group differences were statistically significant, pairwise *post-hoc* comparisons were performed using Bonferroni correction to account for multiple testing and control type I error. Categorical variables were compared using Chi-square or Fisher's exact tests.

To explore the potential influence of confounding factors on coronary artery involvement, restricted exploratory logistic regression analyses were performed adjusting separately for age, sex, complete vs. incomplete Kawasaki disease, fever duration before diagnosis, and time to IVIG treatment. Because only 20 patients exhibited coronary artery involvement and only five patients demonstrated IVIG resistance, fully adjusted multivariable models were not constructed to minimize the risk of model overfitting.

In addition, general linear models were performed to evaluate whether associations between study period and inflammatory markers (CRP and ESR) remained significant after adjustment for age, sex, complete vs. incomplete Kawasaki disease, and fever duration before diagnosis.

Exploratory subgroup analyses according to complete vs. incomplete Kawasaki disease were additionally performed using Fisher-Freeman-Halton exact testing because of small expected cell counts. A *p* value < 0.05 was considered statistically significant.

## Results

### Patient characteristics

**Figure 1:**
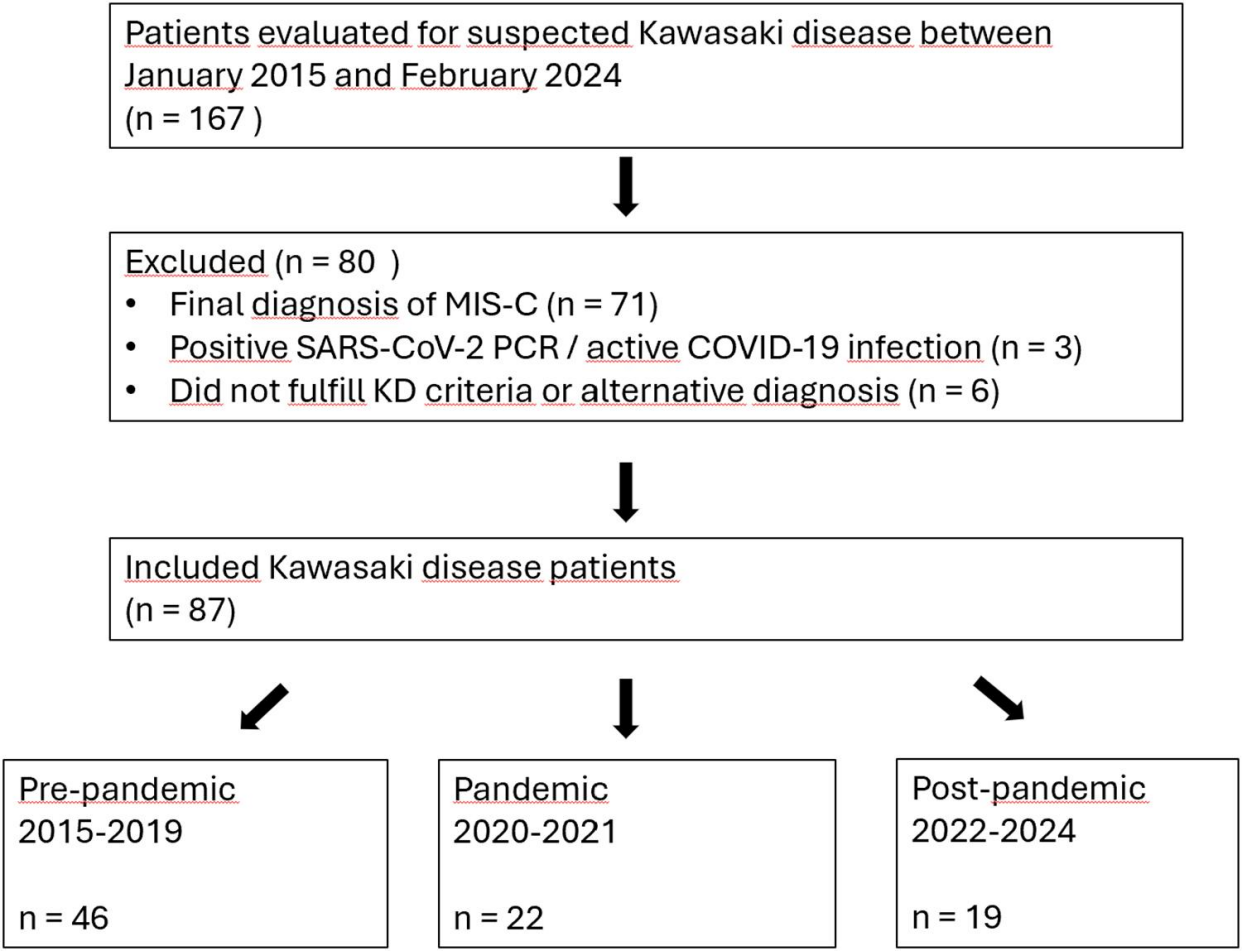
Flow diagram of patient selection and cohort formation.

**Figure 2:**
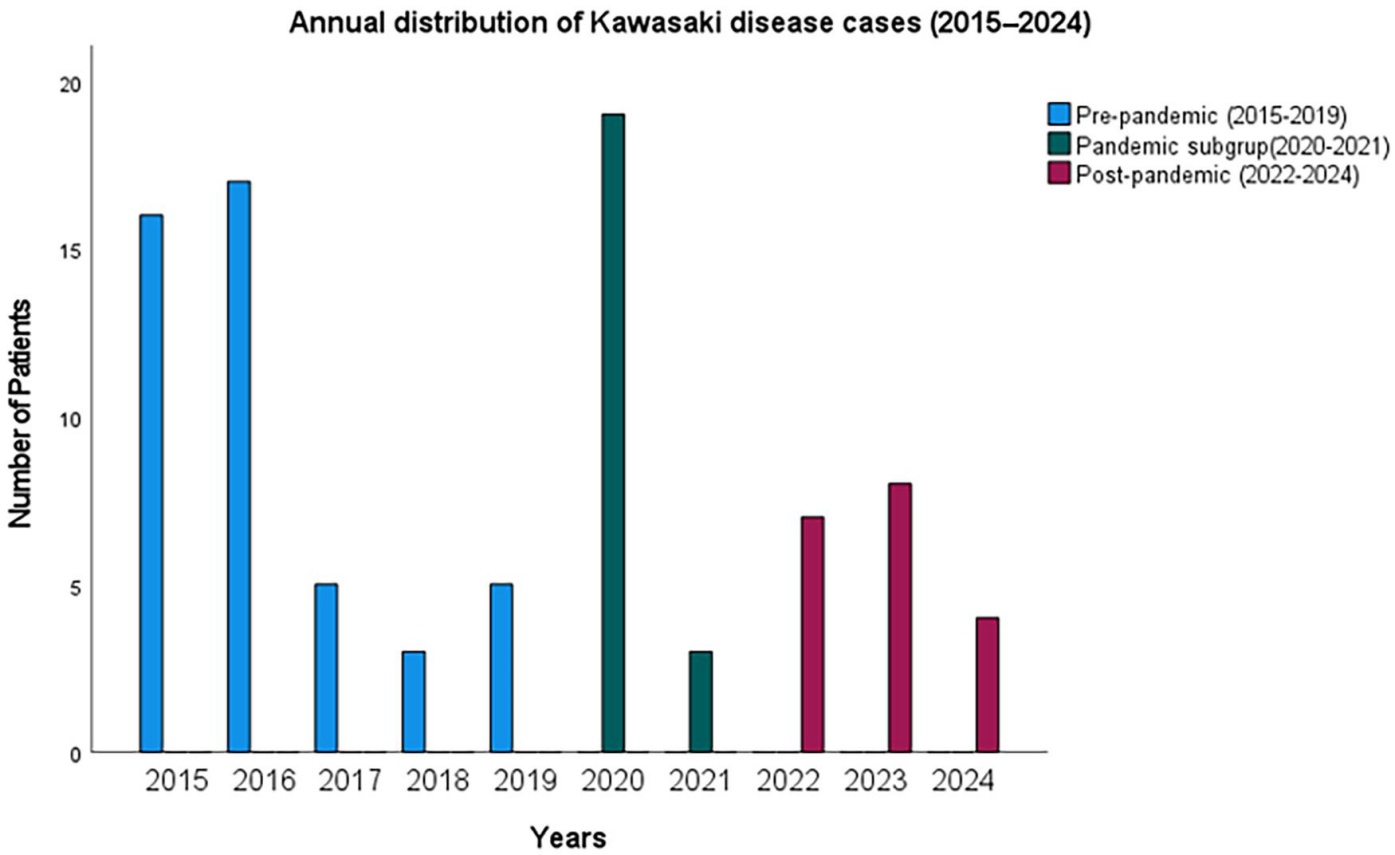
Annual distribution of Kawasaki disease cases from 2015 to 2024 according to study period. Patients were classified into three groups: the pre-pandemic period (2015–2019), the pandemic period (2020–2021), and the post-pandemic period (2022–2024).

A total of 87 children diagnosed with Kawasaki disease were included in the study: 46 in the pre-pandemic period (2015–2019), 22 during the pandemic (2020–2021), and 19 in the post-pandemic period (2022–2024). The patient selection process and reasons for exclusion are summarized in Figure 1. More than 80% of pandemic-period cases (19 of 22 patients) occurred within the first seven months (March–September 2020), followed by a marked decline thereafter (Figure 2).

**Figure 3:**
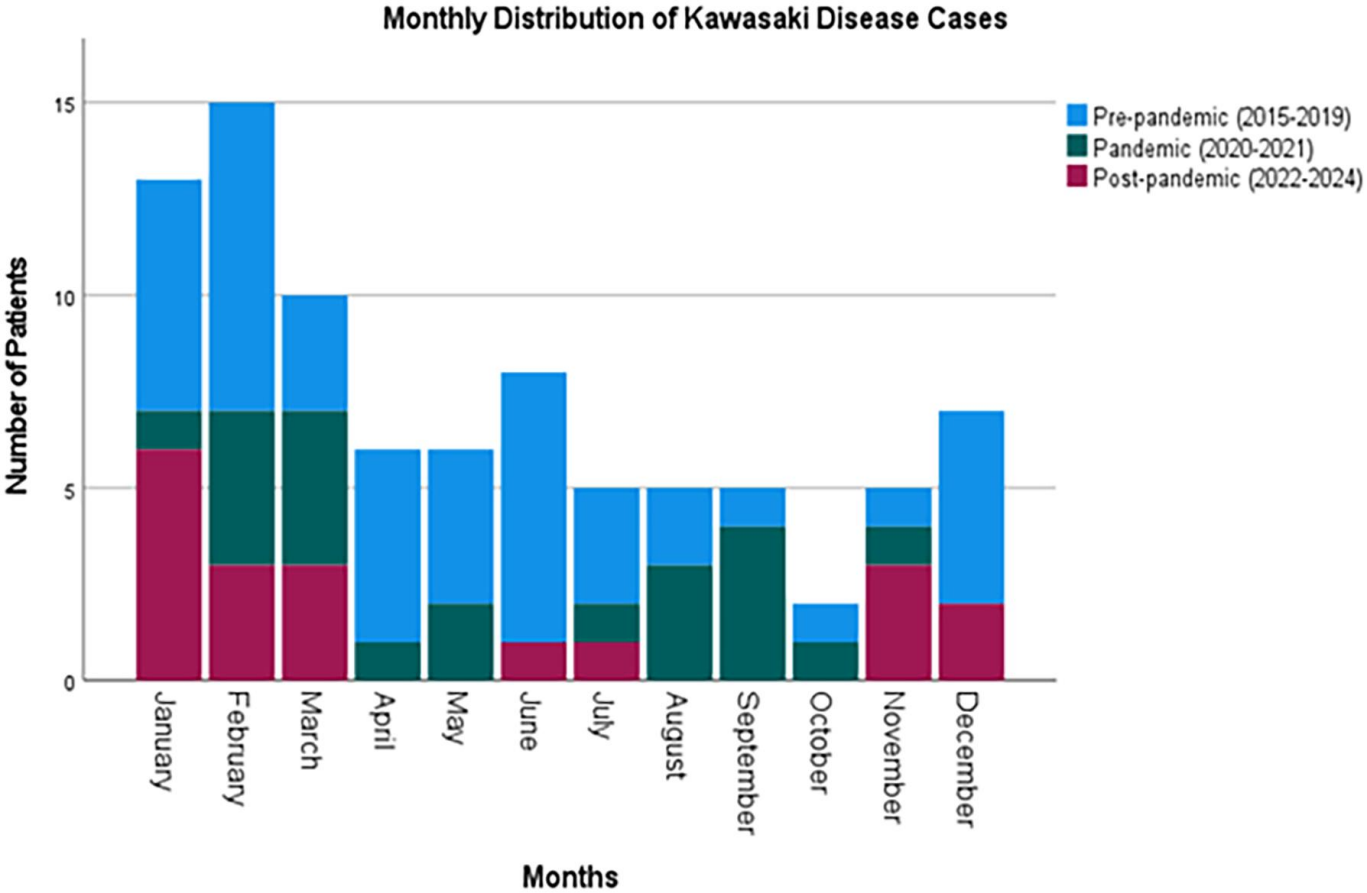
Monthly distribution of Kawasaki disease cases across pre-pandemic, pandemic, and post-pandemic periods (2015–2024).

Monthly analysis of Kawasaki disease cases showed variation across the three defined study periods (Figure 3). In the pre-pandemic period, the highest number of diagnoses occurred between January and March, with a gradual decline during spring and summer months. During the pandemic period, case counts peaked again in February and March, while the remaining months demonstrated a more irregular pattern. In the post-pandemic period, cases were again most frequently observed in winter months, particularly in January and December. Across all three periods, case numbers were lowest between June and August, with a slight increase observed during autumn months.

Age, sex distribution, and the proportion of patients younger than 1 year were comparable across the three periods (all *p* > 0.05).

### Clinical features

The frequency of incomplete Kawasaki disease was numerically higher during the pandemic period, but the difference did not reach statistical significance (*p* = 0.061). The duration of fever before diagnosis and the frequencies of conjunctivitis, oral mucosal changes, rash, and extremity changes were similar among the three periods (all *p* > 0.05).

The frequency of cervical lymphadenopathy differed significantly among periods (*p* < 0.001), being lowest during the pandemic period and higher in the pre-pandemic and post-pandemic groups (Table 1).

**Table 1:** Baseline demographic, clinical, laboratory, and diagnostic characteristics of patients with Kawasaki disease according to study period.

Variable	Pre-pandemic (2015–2019) (*n* = 46)	Pandemic (2020–2021) (*n* = 22)	Post-pandemic (2022–2024) (*n* = 19)	*p* value
Age (months)	38.5 ± 29.0	32.9 ± 35.9	42.4 ± 27.1	0.223
Male sex, *n* (%)	29 (63)	9 (40)	13 (68)	0.137
Age <1 year, *n* (%)	7 (15.2)	6 (27.3)	2 (10.5)	0.319
Clinical features				
Incomplete form, *n* (%)	30 (65.2)	17 (77.3)	8 (42.1)	0.061
Fever duration before diagnosis, median (IQR), days	7.0 (6–9.25)	7.0 (6–8)	7.0 (5–8)	0.332
Conjunctivitis, *n* (%)	25 (54.3)	15 (68.2)	16 (84.2)	0.067
Oral mucosal changes, *n* (%)	29 (63.0)	17 (77.3)	17 (89.5)	0.080
Rash, *n* (%)	28 (60.9)	16 (72.7)	12 (63.2)	0.344
Extremity changes, *n* (%)	15 (32.6)	11 (50.0)	10 (52.6)	0.210
Cervical lymphadenopathy, *n* (%)	31 (67.4)^a^	4 (18.2)^b^	15 (78.9)^a^	<0.001
Laboratory findings at diagnosis, mean ± SD, median (interquartile range, IQR) or *n* (%)				
WBC (×10⁹/L)	15.1 ± 8.1	16.3 ± 6.6	14.9 ± 6.5	0.565
Hemoglobin (g/dL)	10.2 ± 1.3	10.1 ± 1.1	10.8 ± 1.1	0.124
Platelet count (×10⁹/L)	455 ± 233	566 ± 214	503 ± 170	0.145
AST (IU/L), median (interquartile range, IQR)	40 (30–55)	44 (33–67)	53 (42–72)	0.155
ALT (IU/L), median (interquartile range, IQR)	42 (28–55)	45 (31–61)	48 (33–70)	0.727
Albumin (g/dL)	3.44 ± 0.49	3.39 ± 0.52	3.70 ± 0.49	0.099
CRP (mg/L)	87 ± 73^a^	134 ± 59^b^	91 ± 39^a^	0.012
ESR (mm/h)	67.8 ± 30.0^a^	87.3 ± 25.8^b^	76.2 ± 21.4^a^	0.026
Anemia, *n* (%)	27 (58.7)	17 (77.3)	8 (42.1)	0.071
Leukocytosis, *n* (%)	23 (50.0)	12 (54.5)	13 (68.4)	0.397
Thrombocytosis, *n* (%)	26 (56.5)^a^	19 (86.4)^b^	14 (73.7)^ab^	0.040*
Elevated liver enzymes, *n* (%)				
AST elevation	11 (23.9)^a^	7 (31.8)^a^	12 (63.2)^b^	0.010*
ALT elevation	10 (21.7)^a^	6 (27.3)^a^	11 (57.9)^b^	0.015*
Hypoalbuminemia, *n* (%)	8 (17.4)	5 (22.7)	3 (15.8)	0.872

WBC, white blood cell count; AST, aspartate aminotransferase; ALT, alanine aminotransferase; CRP, C-reactive protein; ESR, erythrocyte sedimentation rate; SD, standard deviation; IQR, interquartile range. Data are presented as mean ± SD, median (IQR), or *n* (%). Overall *p* values represent comparisons among the three periods. Superscript letters indicate *post-hoc* pairwise comparisons after Bonferroni correction; groups sharing at least one common letter are not significantly different, whereas groups with different letters differ significantly. A *p* value <0.05 was considered statistically significant.

* Threshold-based categorical definitions may yield different results from continuous comparisons.

### Laboratory findings at diagnosis

Mean WBC counts were similar across periods (15.1 ± 8.1 × 10^9^/L in the pre-pandemic group, 16.3 ± 6.6 × 10^9^/L during the pandemic period, and 14.9 ± 6.5 × 10^9^/L in the post-pandemic period; *p* = 0.565). Likewise, hemoglobin, albumin, and continuous transaminase values did not differ significantly among groups.

Inflammatory markers demonstrated clear between-group differences. Mean CRP levels were 87 ± 73 mg/L in the pre-pandemic period, 134 ± 59 mg/L during the pandemic period, and 91 ± 39 mg/L in the post-pandemic period, with a statistically significant overall difference (*p* = 0.012). *post-hoc* analyses demonstrated that CRP levels were significantly higher during the pandemic period than during both the pre-pandemic and post-pandemic periods. Mean ESR values were 67.8 ± 30.0, 87.3 ± 25.8, and 76.2 ± 21.4 mm/h across the three periods, respectively, with a significant overall difference (*p* = 0.026); however, pairwise comparisons were not significant after Bonferroni correction. To further evaluate the observed differences in inflammatory markers, adjusted general linear models including age, sex, complete vs. incomplete Kawasaki disease, and fever duration before diagnosis were performed. Study period remained independently associated with higher CRP levels after adjustment (*p* = 0.017), whereas the association with ESR was no longer statistically significant (*p* = 0.119) (Supplementary Table S1).

The frequency of thrombocytosis (platelet count ≥450,000/mm^3^) differed significantly across periods (56.5%, 86.4%, and 73.7% in the pre-pandemic, pandemic, and post-pandemic groups, respectively; *p* = 0.040), with *post-hoc* analyses showing that thrombocytosis was more common during the pandemic period than during the pre-pandemic period. Absolute platelet counts were 455 ± 233, 566 ± 214, and 503 ± 170 × 10^9^/L in the pre-pandemic, pandemic, and post-pandemic groups, respectively (*p* = 0.145).

Transaminase elevations were more frequent in the post-pandemic period than in the other two groups. Median AST values were 40 (IQR 30–55), 44 (IQR 33–67), and 53 (IQR 42–72) IU/L, whereas median ALT values were 42 (IQR 28–55), 45 (IQR 31–61), and 48 (IQR 33–70) IU/L in the pre-pandemic, pandemic, and post-pandemic periods, respectively. Despite similar continuous transaminase values, the frequency of AST elevation (>40 IU/L) was 23.9%, 31.8%, and 63.2% (*p* = 0.010), while the frequency of ALT elevation was 21.7%, 27.3%, and 57.9% (*p* = 0.015) across the three periods. (Table 1). Although categorical transaminase elevations differed among periods, continuous AST and ALT values were comparable, suggesting possible threshold-related distributional differences.

### Disease course, treatment, and outcomes

The number of clinical findings at presentation differed among groups (*p* = 0.018), with higher values in the post-pandemic group than in the pandemic group on *post-hoc* analysis (Table 2).

**Table 2:** Clinical outcomes, treatment response, timing metrics, and echocardiographic findings according to study period.

Variable	Pre-pandemic (2015–2019) (*n* = 46)	Pandemic (2020–2021) (*n* = 22)	Post-pandemic (2022–2024) (*n* = 19)	*P* value
Number of clinical findings, median (IQR)	3.0 (2–4)^ab^	3.0 (2–3.25)^b^	4.0 (3–4)^a^	0.018
Day to diagnosis, median (IQR)	2.0 (1–4)^b^	1.0 (1–3)^ab^	1.0 (1–1)^a^	0.001
Time to IVIG treatment, median (IQR)	7 (6–10)	7 (6–8)	6 (5–7.5)	0.173
IVIG treatment, *n* (%)				0.080
Responsive	44 (95.7)	22 (100)	16 (84.2)	
Resistant	2 (4.3)	0	3 (15.8)	
Length of hospital stay (days), median (IQR)	8.0 (4–11)	9.5 (7–11.25)	7.0 (5–9)	0.183
Coronary involvement, *n* (%)	9 (19.6)	8 (36.4)	3 (15.8)	0.243
Dilatation	5	7	2	
Aneurysms	4	1	1	
Small	–	–	1	
Medium	2	–	–	
Giant	2	1	–	
Valvular regurgitation, *n* (%)	10 (21.7)	4 (18.2)	4 (21.1)	0.943

IVIG, intravenous immunoglobulin, IQR, interquartile range. Values are expressed as median (interquartile range, IQR) for continuous variables and number (percentage) for categorical variables. A *p* value < 0.05 was considered statistically significant. Overall *p* values represent comparisons among the three periods. Superscript letters indicate *post-hoc* pairwise comparisons after Bonferroni correction; groups sharing at least one common letter are not significantly different, whereas groups with different letters differ significantly.

Day to diagnosis varied among periods (*p* = 0.001); *post-hoc* analysis showed shorter intervals in the post-pandemic group compared with the pre-pandemic group. Time to IVIG treatment was comparable across the three study periods, with median (IQR) values of 7 (6–10) days in the pre-pandemic group, 7 (6–8) days during the pandemic period, and 6 (5–7.5) days in the post-pandemic period (Kruskal–Wallis *p* = 0.173).

IVIG resistance rates were low across all three periods (*p* = 0.080), and hospitalization duration remained similar throughout the study period (*p* = 0.183).

Coronary artery involvement was recorded in 19.6%, 36.4%, and 15.8% of patients in the pre-pandemic, pandemic, and post-pandemic periods, respectively, with no statistically significant difference among groups (*p* = 0.243). Most abnormalities were mild (dilatation or small aneurysms), and medium or giant aneurysms were uncommon. Valvular regurgitation rates were similar across the three periods (*p* = 0.943). *In restricted exploratory logistic regression analyses adjusting separately for age, sex, complete* vs. *incomplete Kawasaki disease, fever duration before diagnosis, and time to IVIG treatment, study period was not independently associated with coronary artery involvement in any model* (overall model *p*-values ranging from 0.198 to 0.428) (Supplementary Table S2). Similarly, in exploratory subgroup analyses stratified by disease completeness, no significant differences in coronary artery involvement across study periods were observed within either the complete KD subgroup (*p* = 0.379) or the incomplete KD subgroup (*p* = 0.372) (Supplementary Table S3).

## Discussion

Early European reports described an apparent surge in Kawasaki disease (KD)–like cases during the initial months of the COVID-19 pandemic, prompting speculation about a possible link to SARS-CoV-2 infection (5, 6). In contrast, subsequent population-based studies demonstrated a 30%–40% reduction in KD incidence compared with pre-pandemic years (11, 12). This decline was largely attributed to widespread mask use, social distancing, and school closures, which curtailed respiratory viral circulation and supported the concept of infectious triggers in KD pathogenesis (13, 14).

In the present study, nearly half of all cases diagnosed during the pandemic period occurred within the first seven months of the pandemic (March–September 2020), followed by a marked decline in subsequent years. This transient increase resembles the early spike reported by Ünlü et al. in Denmark (15), but differs from trends reported in the United States (7) and Japan (8), where a more sustained reduction was observed. Such heterogeneity may reflect differences in healthcare-seeking behavior, diagnostic awareness, public health measures, and regional circulation of infectious triggers during the pandemic. When all study periods were considered together, our findings suggest a transient early-pandemic increase rather than a sustained post-pandemic rise.

With respect to demographic characteristics, unlike Japanese and Korean reports that noted a relative increase in infants younger than one year during the pandemic (11, 14), our study found no significant shift in age distribution. The male predominance remained stable, aligning with established epidemiologic trends of KD (16, 17). These findings suggest that the demographic profile of children diagnosed with Kawasaki disease in our referral population remained largely unchanged across study periods.

Regarding clinical presentation, the proportion of incomplete Kawasaki disease was numerically higher during the pandemic period in our cohort, although this difference did not reach statistical significance. A similar trend was reported in a recent multicenter study from the United Arab Emirates (9), which found a higher rate of incomplete presentations during the COVID-19 era. In contrast, the number of clinical findings at presentation was higher in the post-pandemic period. This pattern may reflect earlier recognition of febrile inflammatory illness before the full constellation of Kawasaki disease features had evolved during the pandemic, whereas the post-pandemic period may represent a return toward more typical clinical presentations after normalization of public health measures, healthcare utilization, and infectious exposures.

Comparative analyses from different countries have yielded variable results regarding Kawasaki disease phenotypes before and after the pandemic. Some investigators reported reduced frequencies of cervical lymphadenopathy and mucosal changes during the pandemic, together with a relative increase in incomplete presentations (8, 9, 18, 19). The significantly lower frequency of cervical lymphadenopathy observed during the pandemic period in our cohort is consistent with findings reported by others. Alfalasi et al. documented a significant reduction in cervical lymphadenopathy among post-COVID-19 KD patients compared with the pre-pandemic period (50% vs. 72%, *p* = 0.009), and similarly observed a higher proportion of incomplete KD presentations in the post-pandemic era (9). Ding et al. likewise reported a significant decrease in cervical lymphadenopathy, oral mucosal changes, and extremity findings among KD patients diagnosed during the COVID-19 isolation period, and attributed these changes to a concurrent reduction in community viral pathogen circulation (20). The suppression of respiratory viral pathogens through mask use, social distancing, and school closures during the pandemic is widely thought to have reduced infectious triggers for KD; the parallel reduction in cervical lymphadenopathy may reflect a shift in the spectrum of co-circulating pathogens known to elicit prominent lymphadenopathy as part of the KD phenotype. The recovery of cervical lymphadenopathy frequency in our post-pandemic cohort, approaching pre-pandemic levels, further supports the notion that pandemic-era public health measures — rather than a fundamental change in disease biology — were responsible for the altered clinical presentation during the pandemic period.

Laboratory findings showed clear variation across the three study periods. The pandemic period was marked by significantly higher C-reactive protein (CRP) levels, an overall increase in erythrocyte sedimentation rate (ESR), and a higher frequency of thrombocytosis compared with the pre-pandemic period, indicating a stronger inflammatory profile during this phase. Importantly, the association between study period and CRP levels remained significant after adjustment for age, sex, complete vs. incomplete Kawasaki disease, and fever duration before diagnosis. Reports from various regions have described heterogeneous inflammatory patterns during the pandemic, with some studies reporting lower or unchanged levels of acute-phase reactants, including CRP and ESR, compared with the pre-pandemic period (9, 21). These differences may reflect regional variation in infectious exposures, referral patterns, timing of presentation, and differences in case definitions or exclusion of overlapping inflammatory syndromes such as MIS-C.

The post-pandemic period showed a higher frequency of liver transaminase elevations than both the pre-pandemic and pandemic periods. The higher frequency of liver transaminase elevations in the post-pandemic period has not been consistently reported in prior comparative studies. Alfalasi et al. found no significant differences in liver enzyme levels between pre- and post-COVID-19 KD cohorts, while Ünlü et al. reported elevated transaminases specifically during, rather than after, the pandemic period. The underlying reasons for this finding in our cohort remain uncertain. The post-pandemic resurgence of multiple pediatric viral pathogens — including respiratory syncytial virus, adenovirus, and other respiratory viruses — following their suppression during pandemic-era public health measures, has been well documented (22). This rebound, partly attributed to immune debt accumulated during prolonged periods of reduced viral exposure, may have contributed to a higher burden of concurrent viral hepatic involvement in children presenting with Kawasaki disease during the post-pandemic period. However, as the etiology of transaminase elevations was not specifically investigated in this study, these explanations remain speculative, and the small sample size limits definitive conclusions.

Although inflammatory markers were higher during the pandemic period, IVIG resistance rates were similar across the three study periods, suggesting that increased inflammatory burden was not accompanied by a higher frequency of refractory disease in our cohort. Earlier recognition may have contributed to these findings, particularly given the shorter time to diagnosis observed in the post-pandemic period. Notably, time to IVIG treatment was also comparable across study periods, indicating that treatment timing was unlikely to have substantially influenced the observed differences in clinical and inflammatory features.

With regard to hospitalization, Yu et al. reported shorter hospital stays during the pandemic period and attributed this finding to early discharge strategies aimed at reducing the risk of hospital-acquired infections (13). In contrast, length of hospital stay was similar across the three study periods in our cohort, suggesting no major change in hospitalization patterns over time.

Coronary artery involvement did not differ significantly across the study periods; however, numerically higher rates were observed during the pandemic period. These abnormalities were predominantly mild, consisting mainly of coronary dilatation or small aneurysms, whereas medium and giant aneurysms remained uncommon throughout all periods. Previous studies evaluating coronary outcomes in Kawasaki disease during the COVID-19 era have likewise not demonstrated a consistent increase in overall coronary involvement or aneurysm rates (13, 14). Nevertheless, our findings should be interpreted cautiously, as the limited sample size may have reduced the ability to detect clinically meaningful differences between groups.

Consistent with these findings, none of the IVIG-resistant patients across any study period required escalation to corticosteroids or biologic agents, further suggesting no clear worsening of treatment response in this cohort.

### Limitations

This study has several limitations. First, its retrospective single-center design may limit the generalizability of the findings beyond our referral population. Second, the relatively small sample size may have limited statistical power, particularly for detecting modest differences and for subgroup analyses, and may have been insufficient to identify statistically significant differences in low-frequency outcomes such as coronary artery involvement. Furthermore, the limited number of coronary artery involvement events and IVIG-resistant cases precluded the construction of fully adjusted multivariable models. Third, temporal changes in clinical awareness, referral patterns, and healthcare-seeking behavior during the pandemic may have influenced case presentation across study periods. Although fever duration before diagnosis and time to IVIG treatment were comparable across the three study periods, suggesting no major diagnostic or treatment delays, broader changes in healthcare access, referral pathways, and parental healthcare-seeking behavior as contributing factors cannot be fully excluded. Fourth, although echocardiographic assessments were performed by experienced pediatric cardiologists using standardized measurement methods, echocardiographic images were not independently re-evaluated through a centralized core laboratory; therefore, some degree of inter-observer variability and measurement bias cannot be completely excluded. Finally, although extensive clinical and laboratory criteria were applied to minimize overlap with MIS-C, systematic SARS-CoV-2 serologic testing was not available for all patients. Therefore, prior asymptomatic or unrecognized SARS-CoV-2 infection cannot be fully excluded.

## Conclusion

This single-center study suggests that the COVID-19 pandemic was associated with temporal variation in the clinical and inflammatory features of Kawasaki disease. During the pandemic period, inflammatory markers were higher and some clinical features were less frequent, whereas the post-pandemic period was characterized by earlier diagnosis, a higher number of clinical findings at presentation, and more frequent liver transaminase elevations. No clear between-group differences were identified in IVIG resistance, hospitalization duration, or coronary artery involvement. These findings suggest that pandemic-related factors may have influenced disease presentation, although they should be interpreted in the context of the retrospective design and limited sample size.

## Data Availability

The raw data supporting the conclusions of this article will be made available by the authors, without undue reservation.
