# Supplementary material for: Temporal trends in clinical and inflammatory features of Kawasaki disease across the COVID-19 pandemic: a single-center experience from Turkey
Source: Front Pediatr. 2026 Jul 15;14:1871138. doi: 10.3389/fped.2026.1871138 (PMC13416352; doi:10.3389/fped.2026.1871138)
Supplement: Supplementary file 1 [file Supplementaryfile1.docx]

**Suplementary Table 1**. Adjusted general linear models evaluating the association between study period and inflammatory markers

| **Outcome** | **Variable** | **Type III Sum of Squares** | **df** | **F** | **P value** |
| --- | --- | --- | --- | --- | --- |
| **CRP** | Study period | 339.265 | 2 | 4.293 | 0.017 |
|  | Age | 37.944 | 1 | 0.960 | 0.330 |
|  | Fever duration before diagnosis | 0.420 | 1 | 0.011 | 0.918 |
|  | Sex | 25.086 | 1 | 0.635 | 0.428 |
|  | Complete vs. incomplete KD | 85.431 | 1 | 2.162 | 0.146 |
| **ESR** | Study period | 3590.838 | 2 | 2.194 | 0.119 |
|  | Age | 364.058 | 1 | 0.445 | 0.507 |
|  | Fever duration before diagnosis | 0.028 | 1 | <0.001 | 0.995 |
|  | Sex | 41.797 | 1 | 0.051 | 0.822 |
|  | Complete vs. incomplete KD | 505.884 | 1 | 0.618 | 0.434 |
| CRP, C-reactive protein; ESR, erythrocyte sedimentation rate; KD, Kawasaki disease. | | | | | |

**Suplementary Table 2.** Restricted exploratory logistic regression analyses evaluating the association between study period and coronary artery involvement

| **Model** | **Covariates Included** | **Overall p-value for Study Period** | **Pandemic vs. Pre-pandemic OR (95% CI)** | **p-value** | **Post-pandemic vs. Pre-pandemic OR (95% CI)** | **p-value** |
| --- | --- | --- | --- | --- | --- | --- |
| Model 1 | Age | 0.428 | 1.13 (0.26–4.97) | 0.873 | 2.30 (0.48–11.14) | 0.300 |
| Model 2 | Sex | 0.245 | 1.30 (0.31–5.44) | 0.722 | 3.05 (0.66–14.16) | 0.155 |
| Model 3 | Complete vs. incomplete KD | 0.412 | 0.98 (0.22–4.35) | 0.976 | 2.08 (0.43–10.11) | 0.363 |
| Model 4 | Fever duration before diagnosis | 0.198 | 1.11 (0.26–4.79) | 0.893 | 2.92 (0.64–13.35) | 0.166 |
| Model 5 | Time to IVIG treatment | 0.270 | 1.02 (0.23–4.49) | 0.976 | 2.51 (0.54–11.71) | 0.242 |
| KD, Kawasaki disease; OR, odds ratio; CI, confidence interval. | | | | | | |

**Suplementary Table 3.** Exploratory subgroup analyses of coronary artery involvement according to study period

| **Subgroup** | **Pre-pandemic n/N (%)** | **Pandemic n/N (%)** | **Post-pandemic n/N (%)** | **P value** |
| --- | --- | --- | --- | --- |
| Incomplete KD | 7/30 (23.3%) | 7/17 (41.2%) | 3/8 (37.5%) | 0.372 |
| Complete KD | 2/16 (12.5%) | 1/5 (20.0%) | 0/11 (0.0%) | 0.379 |
| KD, Kawasaki disease.  P values were calculated using the Fisher-Freeman-Halton exact test because of small expected cell counts. | | | | |
